# Effects of farnesol and lyticase on the formation of *Candida albicans* biofilm

**DOI:** 10.14202/vetworld.2020.1030-1036

**Published:** 2020-06-08

**Authors:** Nadezhda Sachivkina, Ekaterina Lenchenko, Dmitri Blumenkrants, Alfia Ibragimova, Olga Bazarkina

**Affiliations:** 1 Department of Microbiology and Virology, RUDN University, Miklukho Maklaya Street, 6, Moscow 117198, Russia; 2 Department of Veterinary Medicine, Moscow State University of Food Production, Volokolamskoe Highway, 11, Moscow 125080, Russia; 3 Department of Foreign Languages, Agrarian Technological Institute, RUDN University, Miklukho Maklaya Street, 6, Moscow 117198, Russia; 4 Department of Management and Economy in Pharmacy, Medical Institute, RUDN University, Miklukho Maklaya Street, 6, Moscow 117198, Russia

**Keywords:** antimycotic activity, biofilms, *Candida albicans*, enzyme activities, farnesol, hemolytic activity, HiCrome Candida Agar, lyticase, optical density, quorum sensing

## Abstract

**Background and Aim:**

*Candida albicans* is a dimorphic fungus that has both yeast and filamentous forms. It is part of the normal flora in the oral and genital areas of mammals. One factor for the pathogenicity of *C. albicans* is its ability to switch from yeast to hyphae. The hyphal form adheres and penetrates tissues more readily than the yeast form and produces biofilms that are associated with chronic infection. Biofilms are protective niches that enable microorganisms to be more resistant to antibiotic treatment, thus allowing for persistent infection. The first stage in the transition from yeast to hyphae involves the formation of a germ tube, and this transition is triggered by interactions with host cells. Germ tube formation is dependent on serum, pH, temperature, and quorum-sensing molecules (QSMs). Farnesol, which is a QSM in *C. albicans*, can prevent yeast to hyphae conversion and inhibits the growth of fungal biofilm. Lyticase is a synergistic enzyme complex that catalyzes yeast cell lysis by b-1,3-glucanase and is a highly specific alkaline protease that produces protoplasts or spheroplasts. This study investigated the effect of farnesol and lyticase on the formation of *C. albicans* biofilms.

**Materials and Methods:**

*C. albicans* ATCC 2091 was cultivated on liquid and solid Sabouraud media. The presence of *C. albicans* was confirmed using HiCrome Candida Agar chromogenic medium. Enzyme activities were assayed using a HiCandida Identification Kit. The morphology and densitometry parameters of *C. albicans* biofilms were considered in the presence of farnesol (Sigma-Aldrich, Germany), lyticase (from *Arthrobacter luteus*; Sigma-Aldrich, Germany), and farnesol–lyticase.

**Results:**

This study shows that both farnesol and lyticase possess antifungal activity against *C. albicans* biofilms. A significant difference among treatment groups (p<0.05) was observed from strong biofilm production to medium and weak.

**Conclusion:**

Many studies have been devoted to the antimicrobial action of farnesol. Bacterial enzyme lyticase is also used to degrade fungal cell walls. Both molecules show substantial antifungal properties that are similar to the properties of modern antimycotics. The current study demonstrates that farnesol and lyticase can disrupt biofilm formation in *C. albicans* ATCC 2091, which is an effective biofilm producer.

## Introduction

*Candida albicans* causes disseminated infection in mammals and birds and is also a plant pathogen. It forms mono- and poly-species biofilms in clinical, industrial, and pharmaceutical circumstances [1,2].

The correlation of the morphology and densitometry observations of *Candida* spp. with the synthesis of intercellular matrix and the presence of planktonic cells has been reported in pigs with endogenous infection and in dogs with acute and chronic forms of candidiasis [3,4].

The virulence of *Candida* spp. is a function of adhesiveness, invasiveness, hydrolase production, and dimorphic switching (i.e., the morphological transition from yeast form to a mycelial form). Yeast morphology is required for dissemination, and mycelial morphology is needed for invasion [5-7]. The transcriptional control of adhesion, formation of biofilms, filamentation and production of extracellular polymeric substances, and ability to interact with other species in biofilms *in vivo* determine virulence, protection from immune response, and fungicidal drug resistance [8,9].

The extracellular matrix, dimorphic growth, and presence of *Candida* spp. contribute to the increase in biomass of *Clostridium perfringens* and *Bacteroides fragilis* in cocultured biofilms. In the absence of fungi, bacterial viability decreases. A hyperfilamentous phenotype of *C. albicans* is characterized by high adhesion rates (more than ten-fold) and a significant decrease in dispersion [10]. The hyphal forms of *Candida* spp. provide a hypoxic microenvironment for yeast forms and for *Escherichia coli* [11].

Effective measures for the control and prevention of infectious diseases, including candidiasis, require finding effective antimycotic drugs that can reduce adhesion, disrupt intercellular information exchange, and block synthesis or destroy polymer matrices.

This research focuses on the correlation between the morphologic and densitometric indices of *C. albicans* biofilms and the administration of farnesol and lyticase.

## Materials and Methods

### Ethical approval

This study used a certified strain of *C. albicans* and does not require authorization from the ethics committee.

### Study period and location

These experiments were conducted at the Department of Microbiology and Virology, RUDN University, and Department of Veterinary Medicine, Moscow State University of Food Production, between October 2019 and January 2020.

### Strain

Standardized *C. albican* s strain ATCC 2091 BD Microtrol™ was obtained. Before conducting experiments, the morphological, growth, and enzymatic characteristics were examined to demonstrate phenotypic stability [12].

Morphological and growth properties were observed on liquid and solid Sabouraud media (Biomerieux, France) with glucose, penicillin, and streptomycin (100 ME/L at 25°C, 37°C, 42°C, and 45°C for 24 h). The identity of *C. albicans* was confirmed using HiCrome Candida Agar chromogenic medium (HiMedia, India). Enzyme activities were measured using a HiCandida Identification Kit (HiMedia, India) containing urease, melibiose, lactose, maltose, sucrose, galactose, cellobiose, inositol, xylose, dulcitol, raffinose, and trehalose. Daily cultures of microorganisms with an optical density (OD) of 0.5 at a wavelength of 620 nm (0.5 OD at 620 nm) were added to 96-Well Microplates, MicroLiter and were cultured at 22.5°C for 48 h.

Hemolytic activity used ready-made 5.0% blood agar with sheep erythrocytes (BioMedia, Russia). *C. albicans* culture was transferred to a nutrient medium, and the plates were incubated at 37°C for 24 h. The appearance of clear halos around colonies (a-hemolysis), the greening of the broth (b-hemolysis), or the absence of color (g-hemolysis) was recorded.

The presence of chlamydospores was assessed in terms of growth after the transfer of cells incubated for 24 h in Sabouraud medium on rice agar (API-System R.A.T., France) and after cultivation at 25°C for 24 h. The formation of hyphal germ tubes was assessed in cells cultured in 1.0 ml meat peptone broth (MPB) supplemented with bovine blood serum (Microgen, Russia) at 37°C for 5 h.

The morphological and densitometry of biofilms were considered after treatment with farnesol (Sigma-Aldrich, Germany), lyticase (from *Arthrobacter luteus*; Sigma-Aldrich, Germany), and farnesol+lyticase.

Control studies used MPB (nutrient broth, HiMedia, India). *C. albicans* was grown in nutrient broth at 37°C for 48 h for experiments I–IV:

- I: Culture of *C. albicans*, 4 (McFarland)
- II: Culture of *C. albicans*, 4 (McFarland), farnesol, 100-400 mM
- III: Culture of *C. albicans*, 4 (McFarland), lyticase, 250-1000 units
- IV: Culture of *C. albicans*, 4 (McFarland), farnesol+lyticase.

For microscopic examination, *C. albicans* was cultivated on glass slides placed in Petri dishes with 20.0 ml MPB and 5.0 ml of an 18 h-old culture at a concentration of 105 CFU/ml at 37°C for 48 h. Test samples were fixed with a mixture of alcohol–ether (1:1) for 10 min and stained with a 0.5% methylene blue [13-15]. The absence of yeast and mycelial forms was determined by microscopy using an optical microscope “BioMed MC-1 Stereo” (Russia). A representative sample showed a frequency of occurrence of ≥90.0%.

The OD of biofilms was measured by the degree of binding of crystal violet (HiMedia, India) at a wavelength of 580 nm (OD580) in an Immunochem-2100 microplate photometric analyzer (HTI, USA). The samples were added to the wells of a 96-well plate (Medpolymer, Russia) and were cultured at 37°C for 48 h. Liquid was removed, and wells were washed 3 times with 200 ml of phosphate-buffered saline solution (pH 7.3). At each washing step, the plates were shaken for 5 min. Biofilms were fixed in 150 ml of 96.0% ethanol for 15 min, and wells were dried at 37°C for 20 min. Biofilms were stained with a 0.5% solution of crystal violet at 37°C for 5 min. The liquid was removed from wells, and the wells were rinsed 3 times with 200 µl of phosphate-buffered saline solution (pH 7.3) and then dried. Dye was eluted from adherent cells with 200 µl of 96.0% ethanol for 30 min [5]. Biofilm formation was differentiated by the intensity of biofilm staining:

- OD_s_ ≤OD_c_: Microorganisms that do not produce biofilm
- OD_c_ <OD_s_ ≤ (2 × OD_c_): Low active biofilm producers
- (2 × OD_c_) < OD_s_ ≤ (4 × OD_c_): Moderate biofilm producers
- (4 × OD_c_) < OD_s_: Potent biofilm producers,

where ODc – optical density of control; ODs – optical density of sample.

### Statistical analysis

Experimental data were processed using descriptive and inferential statistics. The means and standard deviations of optical densities and adhesive properties were calculated using Microsoft Excel. The differences between the means of the test samples and controls were assessed using Student’s t-test, and statistical significance was set at p≤0.05.

## Results

### Culturing of yeast like fungi and biochemical test

*C. albicans* showed a typical round or oval yeast shape with a diameter of 1.5-10 mm, and these properties differentiated them from chlamydospores and pseudomycelia. Spherical chlamydospores that were formed by the rounding of terminal filamentous hyphae were seen on native preparations without staining. These chlamydospores had diameters of 7.0-13.0 mm. The filaments of pseudohyphae had no common membrane or septum and were pressed tightly together. The microscopic images of smears stained with methylene blue revealed seedling tubes, which are the precursors of true hyphae. Microorganisms produced turbidity and formed precipitates and films while growing in nutrient broth. On Sabouraud agar with glucose at 25°C, *Candida* formed smooth, convex, white, soft, and consistent colonies. Growth was also observed at 37°C, 42°C, and 45°C (Table-1).

**Table-1:** Phenotypic characteristics of *Candida albicans.*

Morphological characteristics		Colony characteristics									

		Sabouraud dextrose broth			Sabouraud dextrose agar		HiCrome candida agar		Hemolytic activities		
Spherical or oval yeast chlamydospores hyphae pseudohyphae hyphal germ tubes		Microfluid opacity, membrane, incoherent precipitate			Smooth, convex, white, soft texture		Colonies of a light-green color		*γ*-hemolysis		

**Enzyme activity**											

Urease	Melibiose	Lactose	Maltose	Sucrose	Galactose	Cellobiose	Inositol	Xylose	Dulcitol	Raffinose	Trehalose
−	−	−	+	+	+	−	−	+	−	−	+

The studied strain displayed the typical phenotypic characteristics described in the *Guide to Clinically Significant Fungi* by Deanna A. Sutton (2001).

### Optical microscopy

**Figure-1:**
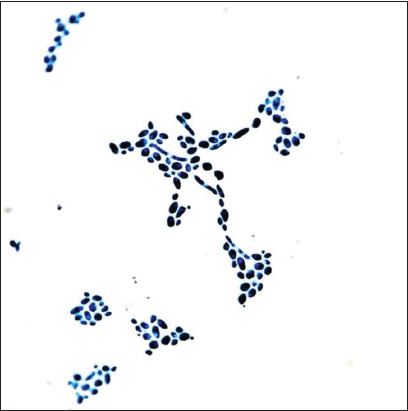
The intensity of *Candida albicans* biofilms, 37°C 48 h – experiment (I): Yeast and mycelial forms forming the aggregation of microorganisms, covered by a thin layer of blue intercellular matrix. Staining with a methylene blue, optical microscopy 200×.

*C. albicans* grown at 37°C for 48 h (experiment I) showed dimorphic fungal growth, adherence to substrate (glass) in heterogeneous structures, vegetative yeast forms, blastospores and hyphae forms, hyphae, and pseudohyphae. Yeast forms were stained intensely blue and elongated hyphae, pseudohyphae, and communicating filaments were also stained with blue. Blastospores were located on the hyphae. Blue chlamydospores with a double membrane were observed on the terminal extensions of the hyphae. The blue and deep blue aggregations of the heterogeneous structures of yeast and hyphae forms were connected by a layer of intercellular matrix (Figure-1).

A biofilm develops through the adhesion, fixation, and synthesis of extracellular polymeric substances that enable intercellular communication. Nutrients accumulate in the population as cells proliferate. An increase in cell population leads to an increase in the density of the extracellular matrix. An increasingly compact matrix “cements” yeast and mycelial forms into branched, separate, round, or oval structures. These structures are separated by matrix voids and are probably filled with liquid.

**Figure-2:**
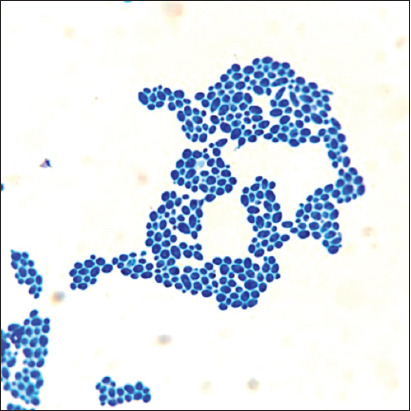
The intensity of the formation of biofilms of *Candida albicans*, 37°C 48 h – experience (I): Isolated structures separated by matrix voids. Staining with a methylene blue, optical microscopy 200×.

**Figure-3:**
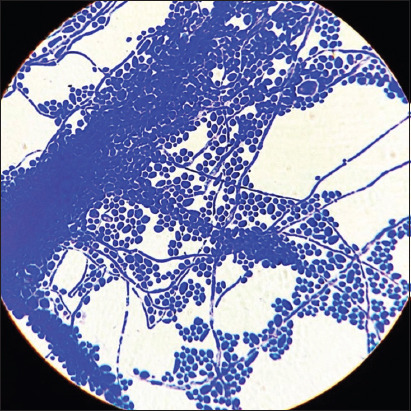
The intensity of the formation of biofilms of *Candida albicans*, 37°C 48 h – experiment (I): Architectonics of a grown biofilm. Coaggregation of yeast and mycelial forms is combined with an exocellular matrix. The long branched hyphal forms are composing dense structures from pseudomycelia. Staining with a methylene blue, optical microscopy 100×.

The heterogeneous structure of the fungal population in this study consisted of yeast forms, hyphal germ tubes, and short hyphae (Figure-2). Microcolonies were presented with aggregates of yeast forms, fermentation tubes, and short hyphae and were combined in a basal layer surrounded by an intercellular polymer matrix. The stable architecture of the 3D biofilm progressed to the coaggregation of yeast and mycelial forms combined with an extracellular matrix. Long-branched hyphal forms were composed of dense pseudomycelial structures (Figure-3). At the center of the oval microcolonies, the matrix became prominent with the absence of separate cells. In the periphery of colonies, the extracellular matrix gradually thinned, and individual cells were arranged in orderly concentric rows.

**Figure-4:**
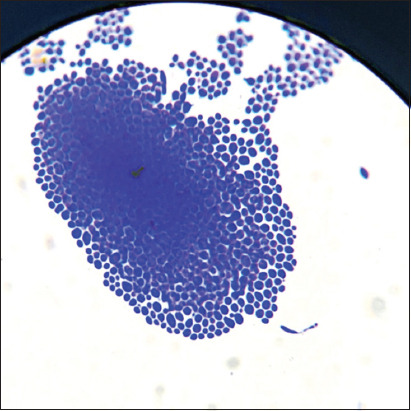
The intensity of the formation of *Candida albicans* biofilms, 37°C 48 h – experiment (I): Microcolony – coaggregation of microorganisms united by an exocellular matrix more pronounced in the central part and much thinner on the periphery. Staining with a methylene blue, optical microscopy 100×.

In areas of thinning, the population was unstructured, and cells displayed different sizes and shapes. Dispersion regions were observed at the periphery of microcolonies. This stage is characterized by the destruction of the intercellular matrix and the subsequent separation of “daughter” yeast cells in branched structures for the colonization of a free substrate (Figure-4).

After exposure to antimycotic agents (experiments II and III), cultures were characterized by the presence of yeast forms, and coadhesive properties were impaired. Hyphal growth was significantly reduced or absent. Single cells had an angular shape, and hyphae were short and collapsed.

Changes in the colony structure of *C. albicans* biofilms were evaluated by the presence (“+”) or absence (“−”) of yeast and mycelial forms (Table-2). A representative sample showed a reliability of ≥90.0% for the observed fields.

**Table-2:** Changes in the colony of *Candida albicans* biofilms after exposure to antifungal drugs.

Groups	Changes in the colony structures (≥90.0% in the microscope POV)		

	Solitary cells and clusters	Microcolonies	Mycelial forms
I – *C. albicans*	+	+	+
II – *C. albicans* – Farnesol, 400 μM	+	–	–
III –*C. albicans* – Lyticase, 1000 units	+	–	–
IV –*C. albicans* – Farnesol 400 μM – Lyticase, 1000 units	+	–	–

+ - presence, – - absence. *Candida albicans=C. albicans*

- Experiment I: Coaggregation of yeast and mycelial forms combined by extracellular matrix and long-branched hyphal forms and formation of dense structures from pseudomycelia
- Experiment II–III: Microcolonies
- Experiment IV: Single cells and clusters.

### Densitometric values

Indicators of OD in experiment I: An OD_s_ of 5.03±0.06 for *C. albicans* incubated under our standard conditions exceeded the control index OD_c_ of 0.098±0.09 more than five-fold. These cells produced substantial biofilm (Table-3). In experiment II, the ODs for cells treated with farnesol (0.382±0.16) exceeded the control OD_c_ of 0.098±0.09 by less than four-fold, thus indicating moderate biofilm production (Table-4). Similarly, for experiment III, the ODs for cells treated with lyticase (0.284±0.08) exceeded the control OD_c_ (0.099±0.08) by less than three-fold, thus indicating moderate biofilm production (Table-5). In the presence of both farnesol and lyticase, OD_s_ (0.196±0.06) exceeded the control OD_c_ (0.098±0.12) by approximately two-fold, thus indicating weak biofilm production (Table-6).

**Table-3:** Analysis of the optical density of biofilms.

Microorganism culture	Optical density (OD)			

	Control (OD_c_)	Exp (OD_s_)	Δ (OD_s_–OD_c_)	Intensity (I)
*Candida albicans*	0.099±0.03	0.503±0.06	0.404±0.09	≥0.4

≤0.1 – non-biofilm producing microorganisms, ≤0.2 – weak biofilm producers, ≤ 0.3 – medium biofilm producers, ≥0.4 – strong biofilm producers

**Table-4:** Analysis of the optical density of biofilms of microorganisms when exposed to the Farnesol.

Medicine concentration, μM	Optical density (OD)			

	Control (OD_c_)	Exp (OD_s_)	Δ (OD_s_–OD_c_)	Intensity (*I*)
100	0.096±0.03	0.395±0.06	0.299±0.09	≤0.3
200	0.099±0.05	0.372±0.03	0.273±0.08	≤0.3
400	0.098±0.01	0.365±0.07	0.267±0.09	≤0.3

≤0.1 – non-biofilm producing microorganisms; ≤0.2 – weak biofilm producers; ≤0.3 – medium biofilm producers; ≥0.4 – strong biofilm producers

**Table-5:** Analysis of the optical density of biofilms of microorganisms when exposed to the Lyticase.

Medicine concentration, units	Optical density (OD)			

	Control (OD_c_)	Exp (OD_s_)	Δ (OD_s_–OD_c_)	Intensity (*I*)
250	0.099±0.08	0.371±0.09	0.272±0.17	≤0.3
500	0.096±0.02	0.335±0.01	0.239±0.03	≤0.3
1000	0.098±0.09	0.299±0.05	0.201±0.14	≤0.3

≤0.1 – non-biofilm producing microorganisms, ≤0.2 – weak biofilm producers, ≤0.3 – medium biofilm producers, ≥0.4 – strong biofilm producers

**Table-6:** Analysis of the optical density of biofilms of microorganisms when exposed to the Farnesol and Lyticase.

Medicine concentration, μM – Farnesol units - Lyticase	Optical density (OD)			

	Control (OD_c_)	Exp (OD_s_)	Δ (OD_s_–OD_c_)	Intensity (*I*)
100 250	0.099±0.02	0.259±0.09	0.160±0.11	≤0.2
200 500	0.098±0.04	0.219±0.01	0.121±0.05	≤0.2
400 1000	0.098±0.07	0.200±0.06	0.102±0.09	≤0.2

≤0.1 – non-biofilm producing microorganisms, ≤0.2 – weak biofilm producers, ≤0.3 – medium biofilm producers, ≥0.4 – strong biofilm producers

*C. albicans* exposed to antimycotic agents showed parallel changes in morphometric (%) and densitometry indicators (OD_s_). The changes included reduced frequency of clusters and OD. The frequency of clusters, which is defined as the aggregation of microorganisms united by a thin layer of intercellular matrix, significantly decreased from high cluster frequency in experiment I to moderate cluster frequency in the presence of farnesol (experiment II) or lyticase (experiment III). The combination of farnesol and lyticase (experiment IV) led to the low cluster frequency.

## Discussion

Our research, along with the previous reports, indicates that various taxonomic groups display common patterns of biofilm formation: Adhesion, fixation, maturation, growth, and dispersion [13]. The biosynthesis of exopolysaccharides is accompanied by a decrease in metabolic activity and the transformation of microbial populations to an “uncultivated condition” [14]. The morphofunctional stability of *C. albicans* biofilms is based on yeast and hyphal forms, and the process of seedling formation ensures the development of the intercellular matrices [15].

The adsorption, adhesion, and fixation of basal yeast layers, along with the early development of hyphae and matrix, contribute to the increased biomass of yeast, hyphae, pseudohyphae, extracellular matrix, and water channels. These structures promote the movement of nutrients and the dissemination of cells. Furthermore, filamentation is directly associated with the increased density of *Candida* spp. ­biofilms [16]. The formation of a heterogeneous structure of *C. albicans* biofilms is a multistage process. A 3D biofilm structure was detected after 38-72 h. The biofilm consisted of a dense network of yeast cells, hyphae, and pseudohyphae surrounded by an intercellular polymer matrix [3,17].

Among the 47 *Candida* spp. isolates, the most common polymorphism primer is OPA 9. The similarity coefficients reached 95.0% for six clusters, including *C. albicans* from vaginal infections. The dispersion of cells from a cluster is the result of shifting that is unrelated to the dispersion caused by a transcription regulator signal [8].

The destruction of the cell wall and the extraction of *C. albicans* DNA are 90 min faster when exposed to recombinant lyticase, and this can be accomplished with mechanical disruption using glass beads [18,19]. Lyticase caused a decrease in OD to 53.0%, a decrease in adhesion to epithelial cells, and an inhibition of mycelial growth [20,21]. Xylitol (10.0%) and sodium alginate (4.16%) suppressed the virulence of *C. albicans* by preventing the formation of hyphal forms and by reducing biofilm density to 47.0-52.0% [14].

When exposed to increasing farnesol concentrations (0, 3, 30, and 300 μM), morphological transition from yeast to hyphal forms was prevented at the highest concentration[22]. When exposed to farnesol at concentrations of 1-50 μmM, a decrease in the pathogenicity of *C. albicans* was noted due to the inhibition of hyphae formation. This inhibitory effect of farnesol was accompanied by a significant increase in culture density, accumulation on cell surfaces, and downregulation of proteins that determine the structural organization of biofilms [23,24]. Farnesol regulates the specific components of signal transmission and transcription, thus inhibiting the hyphal growth of the hyphae of *C. albicans*, affecting the interaction of mRNA with the small ribosomal subunit, and leading to a decrease in the level of the initiating ribosomal complex [15].

Farnesol inhibited the formation of *C. albicans* mycelium but did not reduce the growth of hyphal germ tubes. At a concentration of 450.0 μM, farnesol inhibited biofilm formation by 35.0% [16]. Farnesol (300.0 μM) inhibited the transition of yeast to the hyphal form. Furthermore, due to a decrease in the expression of Sap2 and Sap4–Sap6, the shape of the cells was changed, cell wall integrity was impaired, and increased granulation of the cytoplasm was induced with the presence of large vacuoles [24]. A significant decrease (56.2%) in the biomass of *C. albicans* biofilms was observed at a 12.5% concentration of farnesol. It was found that the presence of the transcription factor “TEC1” protects the biofilm from drug diffusion [25,26].

## Conclusion

We show the coaggregation of yeast and mycelial forms that is united by an exocellular matrix, long-branched hyphal forms, and dense structures formed by pseudomycelia. When exposed to antimycotic agents, cultures were characterized by the predominant presence of yeast forms, impaired coadhesion, and reduced or absent hyphal growth. Single cells displayed an angular shape, and hyphae were short and collapsed. The frequency of clusters united by a thin layer of intercellular matrix significantly decreased in the following order: *C. albicans* (experiment I), prominent biofilm production; farnesol (experiment II) and lyticase exposed cells (experiment III), moderate biofilm production; and farnesol and lyticase (experiment IV), low biofilm production.

## Authors’ Contributions

NS and EL had the original idea for the study and carried out the design. DB collected the samples. AI was responsible for data analysis and data cleaning. EL, OB, and NS drafted the manuscript. The final draft manuscript was revised by all authors. All authors edited, read, and approved the final manuscript.
